# Six-Month Patency of Long Carotid Bypass Grafts Constructed with In-Body Tissue Architecture-Induced Small-Diameter Biotubes in a Goat Model

**DOI:** 10.3390/bioengineering12030260

**Published:** 2025-03-05

**Authors:** Kazuki Mori, Tadashi Umeno, Takayuki Kawashima, Takashi Shuto, Ryosuke Iwai, Lupeng Teng, Tsutomu Tajikawa, Yasuhide Nakayama, Shinji Miyamoto

**Affiliations:** 1 Department of Cardiovascular Surgery, Oita University, Yufu 879-5593, Japan; umenot@oita-u.ac.jp (T.U.); t-kawashima@oita-u.ac.jp (T.K.); shutot@oita-u.ac.jp (T.S.); smiyamot@oita-u.ac.jp (S.M.); 2 Institute of Frontier Science and Technology, Okayama University of Science, Okayama 700-0005, Japan; iwai@ous.ac.jp (R.I.); r23ndv8iu@ous.jp (L.T.); 3 Department of Mechanical Engineering, Faculty of Engineering Science, Kansai University, Osaka 564-8680, Japan; tajikawa@kansai-u.ac.jp; 4 Osaka Laboratory, Biotube Co., Ltd., Osaka 565-0842, Japan; y.nakayama@biotube.co.jp

**Keywords:** in-body tissue architecture technology, tissue-engineered vascular graft, Biotube, regenerative medicine, vascular surgery

## Abstract

This study investigated the long-term patency of regenerative Biotube grafts and discusses their feasibility as an alternative to autologous vein grafts for peripheral artery disease. Six Biotubes with a diameter of 4 mm were autologously fabricated in recipients using in vivo tissue engineering (in-body tissue architecture) technology and implanted as carotid artery bypass grafts in a goat model. All six grafts remained patent at 6 months despite exceeding 10 cm in length, demonstrating their biocompatibility and durability. Histological analysis revealed neointima formation, endothelialization, and minimal inflammation. However, in one goat, a graft developed stenosis, while another showed dilatation. These findings demonstrate the use of Biotubes as a viable option for peripheral vascular reconstruction as tissue-engineered vascular grafts. However, further optimization is needed to address emerging issues with their use, such as stenosis and aneurysm formation, to improve long-term patency.

## 1. Introduction

Peripheral artery disease (PAD) is a significant health condition, particularly in aging populations and individuals with lifestyle-related diseases. Critical limb ischemia (CLI), a severe manifestation of PAD, can lead to limb amputation and thus adversely impact quality of life [1,2,3]. Despite advancements in endovascular interventions, the surgical bypass remains the gold standard for revascularization, especially in the distal lower extremity [3,4,5]. Considerable evidence supports the use of the great saphenous vein as the optimal graft for a lower extremity bypass [4,6,7]. Although alternative grafts to the great saphenous vein have been proposed, unfeasible grafts can limit surgical options [8]. While small-diameter vascular prostheses offer a potential solution, their long-term patency is limited by issues such as thrombosis, biocompatibility, and infection [9,10,11]. Tissue-engineered vascular grafts (TEVGs) are a promising alternative because they can be tailored to specific patient needs and overcome the limitations of synthetic grafts [12,13,14,15,16].

The focal point of our study is the development of in vivo TEVGs by implementing in-body tissue architecture (iBTA) technology [17]. The utilization of iBTA technology is predicated on the exploitation of the encapsulation reaction that occurs in the vicinity of subcutaneously implanted foreign bodies. This approach facilitates the fabrication of custom-shaped autologous collagenous tissue constructs. Collagen-based tubular tissue fragments produced using iBTA technology are called Biotubes [17,18,19].

In this study, we investigated the 6-month outcomes of using Biotubes for a carotid artery bypass in a goat model based on a preliminary study [18], which assessed the efficacy of iBTA-derived Biotubes in the carotid artery bypass. The findings of this study demonstrate the feasibility and potential of using Biotubes to address the unmet need for small-diameter vascular grafts.

## 2. Materials and Methods

### 2.1. Preparation of Biotubes

**Figure 1:**
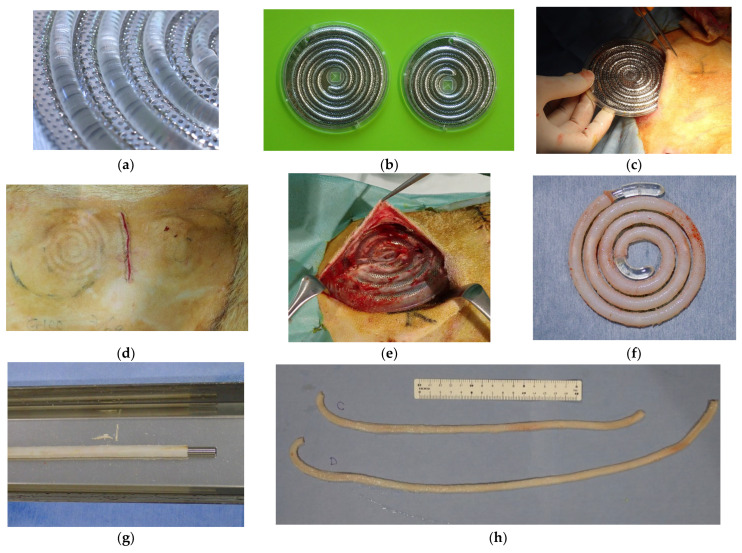
(**a**) A spiral-shaped plastic core rod is positioned between two stainless steel disks, leaving a 0.85 mm gap. The disks are porous and designed to allow fibroblasts to penetrate. (**b**) Two mold sizes were utilized in this study: 86 and 97 mm, inner diameter. The thickness of both was 5.4 mm. (**c**,**d**) Molds were implanted into subcutaneous pockets, with two on each side of the thoracoabdominal region. (**e**) The molds were extracted after 2–3 months of implantation. (**f**) Collagen tissue taking on the shape of the gap around the plastic core was formed after the disk was removed. (**g**) Biotubes, inserted with a straight rod after the removal of the spiral core, were dehydrated and fixed in 70% ethanol. (**h**) Biotubes with lengths of approximately 40 and 55 cm were produced.

Mold preparation and a surgical technique were performed based on previous reports with some modifications [17,18]. The mold consisted of a spiral plastic core rod 4 mm in diameter and two porous stainless disks (Figure 1a). The gap between the core rod and stainless disks was set to 0.85 mm. Tissues containing fibroblasts infiltrated this gap through numerous pores, resulting in the formation of Biotubes. Two mold sizes with diameters of 86 and 97 mm and a thickness of 5.4 mm were prepared (Figure 1b). The molds produced 40- or 55 cm long Biotubes with an inner diameter of 4 mm and a wall thickness of 0.85 mm. The diameter of the Biotube was determined on the basis of the measured diameter of the goat carotid artery. The molds were embedded subcutaneously in the bilateral abdomen of three Saanen goats (age: ≥12 months; weight: 45–55 kg; four molds per animal) under general anesthesia (Figure 1c,d). Anesthesia was induced using 2 mg/kg ketamine and maintained with 2–3% sevoflurane. After 2–3 months, the molds were extracted under general anesthesia as described above (Figure 1e). It was previously demonstrated that a one-month mold embedding period resulted in inadequate Biotube strength and formation [17,18]. Therefore, the embedding period was established as 2–3 months. The Biotubes were carefully extracted from the molds and visually evaluated for formation defects (Figure 1f). The Biotubes were soaked in 70% ethanol for 30 min to dehydrate and fix them. A straight rod was inserted into the graft during fixation in ethanol to prevent the internal diameter from shrinking below 4 mm (Figure 1g). Furthermore, a pressure leak test was performed by connecting one end of a Biotube to a syringe with a pressure gauge and applying 200 mmHg of water pressure to check for leakages or ruptures. Samples 5 mm wide were cut from each Biotube graft and used to determine the tensile strength. The Biotube samples were placed in a portable uniaxial tensile tester (Stency, AcroEdge, Osaka, Japan), and a tensile test was performed. The rupture strength of the Biotube was defined as the load (N) at which the sample was pulled and ruptured. The Biotubes were preserved in 10% ethanol solution until implantation. Just before implantation, the Biotubes were rinsed with normal saline for 10 min (Figure 1h).

### 2.2. Bypass Procedures

The molds were extracted from one side of the abdomen at a time under general anesthesia as described above. Unilateral carotid bypasses were consecutively performed on the same goats from which each Biotube was obtained. Since the surgery was performed in the lateral decubitus position, only the unilateral carotid bypass was performed to avoid acute occlusion due to graft compression. The contralateral carotid bypass using the same procedure was performed on a different day from the preceding one. The bypass was performed as described previously [18].

**Figure 2:**
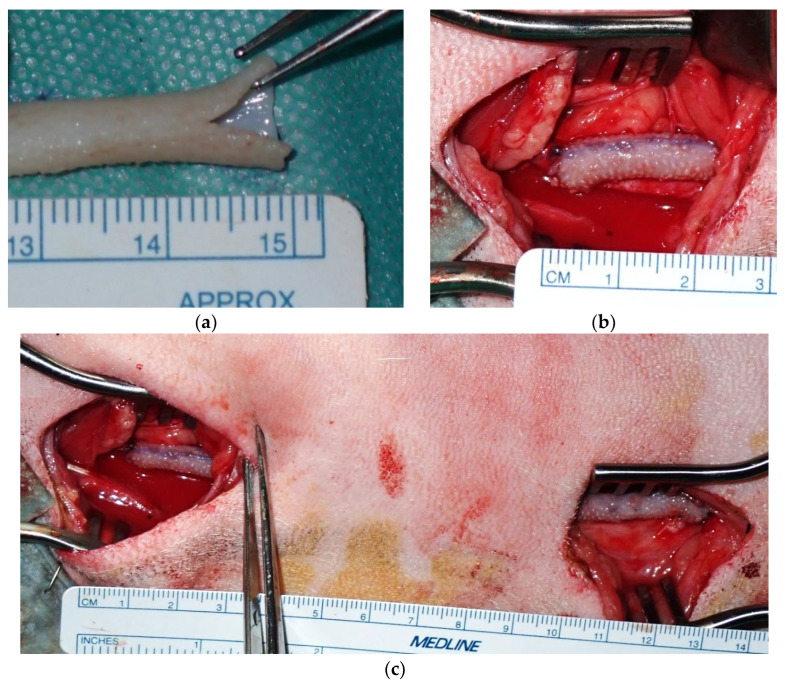
(**a**) A 7 mm longitudinal incision was created in the Biotube. (**b**) End-to-side anastomosis to the carotid artery was achieved using a continuous running 7-0 polypropylene suture. (**c**) A carotid artery bypass was performed using a 12 cm Biotube. The native carotid artery was ligated and transected distal to the proximal anastomosis.

The carotid artery was exposed through two 4 cm skin incisions spaced 7 cm apart. Heparin (300 IU/kg) was administered intravenously, with additional doses administered as needed to maintain an activated clotting time (ACT) ≥ 300 s. End-to-side anastomosis was performed with a 7-0 polypropylene running suture at the proximal side (Figure 2a,b). A 7 mm longitudinal incision was created in the Biotube, and the anastomotic foramen was formed through a 7 mm incision in the artery. Arterial clamping was continued after proximal anastomosis to prevent thrombosis resulting from blood retention in the Biotube. The other end of the Biotube was guided along the native artery to the distal skin incision. Distal anastomosis was also performed using the same technique with a 7-0 polypropylene suture. The artery was de-clamped after distal anastomosis (Figure 2c). At this juncture, the blood had passed through the Biotube for the first time. No clamping of the Biotubes was needed thereafter. The native carotid artery at the proximal site was ligated and sectioned to prevent competition for blood flow. Bypass blood flow was measured with a Transonic HT310 flowmeter (Transonic Systems Inc., Ithaca, NY, USA). After wound closure, the bypass flow was confirmed by vascular ultrasonography. The postoperative medication consisted of dalteparin 1000 IU administered subcutaneously for 1 week, 75 mg clopidogrel, and 100 mg aspirin administered orally for 3 months.

Graft patency was confirmed by vascular ultrasonography. The following variables were evaluated: morphological changes over time from the anastomosis to the entire graft, blood flow in the graft lumen by color Doppler, and blood flow velocity in the graft by pulsed wave Doppler. Blood flow velocity was measured in all cases at the portion of the Biotube, approximately 1 cm peripherally from the proximal anastomosis. Ultrasonography was performed on the day of surgery; 1, 2, 3, and 7 days postoperatively; and weekly thereafter. Morphological evaluation of the implanted Biotubes was performed using contrast-enhanced computed tomography (CT) in addition to ultrasonography 6 months postoperatively. After image evaluation, the Biotubes were harvested for histological evaluation.

### 2.3. Histological Examination

The harvested Biotubes were fixed in 4% paraformaldehyde saline solution (FUJIFILM Wako Pure Chemical Co., Osaka, Japan). The Biotubes were unfolded by longitudinal incision before fixation, and the lumen was macroscopically evaluated along the entire length. These specimens (including the anastomosis) were cut into strips at 1 cm intervals, embedded in paraffin, and sliced into 5 μm sections. The sections were then stained with hematoxylin–eosin, Masson’s trichrome, and Elastica van Gieson at the Institute of Frontier Science and Technology, Okayama University of Science. Furthermore, anti-α-smooth muscle actin (α-SMA) mouse monoclonal antibodies (1:200; 904601, BioLegend, San Diego, CA, USA) were used for immunohistochemical staining. Alexa Fluor^®^ 594 donkey anti-mouse secondary antibodies (1:1000; ab150108, Abcam, Cambridge, UK) were used to assess myofibroblast localization. CD31 rabbit polyclonal antibodies (1:50; ab28364, Abcam) and goat secondary antibodies to rabbit immunoglobulin G (Alexa Fluor^®^ 488, Abcam) were used to confirm vascular endothelial cell localization. Rabbit CD163 monoclonal antibody (1:500; ab182422, Abcam) and goat secondary antibody to rabbit immunoglobulin G (Alexa Fluor^®^ 488) were used to confirm the presence of M2 macrophages. 4′,6-Diamidino-2-phenylindole (DAPI; ProLongTM Gold Antifade Mountant with DAPI, Thermo Fisher Scientific, Inc., Waltham, MA, USA) was used as a nuclear counterstain.

### 2.4. Ethical Approval

All animals were handled in accordance with the Guide for the Care and Use of Laboratory Animals published by the United States National Institutes of Health (publication no. 85-23, revised 1996). This study was approved by the Animal Ethics Committee of Oita University, Japan (Protocol no. 232201).

### 2.5. Statistical Analysis

Continuous variables are expressed as mean ± standard deviation (SD). All statistical analyses were performed using EZR version 1.68 (Saitama Medical Center, Jichi Medical University, Saitama, Japan), which is a graphical user interface for R version 4.2.0 (The R Foundation for Statistical Computing, Vienna, Austria).

## 3. Results

### 3.1. Biotube Production

The molds were embedded into the thoracoabdominal region of three adult goats, with four molds per animal. Biotubes were successfully obtained from all molds after 2–3 months (mean ± SD 10.3 ± 2.2 weeks). No problems were observed in the insertion wounds during implantation. No exudate accumulation or inflammatory findings were observed on removal. All Biotubes formed in the molds were successfully harvested. Leak and pressure tests (200 mmHg) were performed after fixation, and pinhole leaks were found in the two Biotubes. No Biotubes ruptured even under water pressure >200 mm Hg. Two Biotubes found with pinholes also did not rupture, even in areas where no leakage was observed. The Biotubes had a mean tensile strength of 10.98 ± 1.50 N, which was confirmed through the tensile test. Thus, the Biotubes were strong enough to withstand >5 N, the standard strength for a vascular prosthesis according to ISO 7198:2016 [20]. All Biotubes showed similar flexibility to the vein grafts. The Biotubes were tested by bending them while applying water pressure internally, but no occlusion due to kinking was observed.

### 3.2. Bypass Procedure

Bilateral autologous Biotube bypasses (6 bypasses in total) were successfully performed on the three goats. The carotid bypass was performed by selecting the side that exhibited superior strength between the grafts obtained. The Biotubes were durable enough to withstand clamping and suturing during the procedure without sustaining damage. The mean ± SD bypass length was 12.0 ± 0.9 cm. The mean ± SD bypass blood flow was measured at 305.5 ± 69.3 mL/min by the flowmeter. Vascular ultrasonography after wound closure showed a systolic velocity of 39.24 ± 14.26 cm/s.

**Figure 3:**
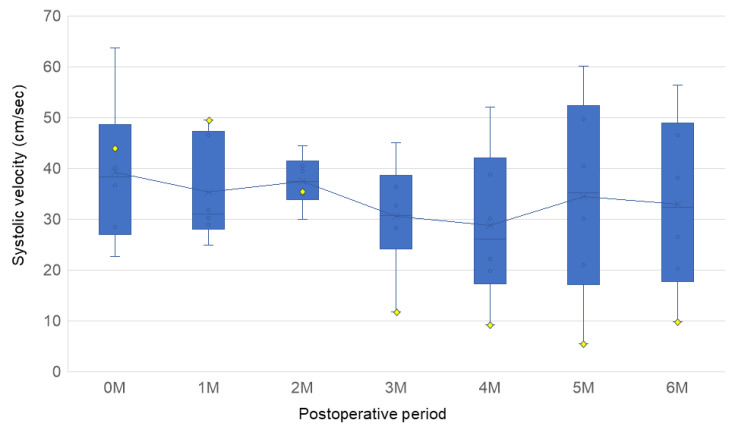
Box plots depicting the distribution of the peak systolic flow velocities, as determined by ultrasound, for each month. Each box represents data from 6 samples (*n* = 6). The yellow diamonds indicate the blood flow velocity in the stenotic bypass. A significant decrease in velocity, up to 11.73 cm/s, was observed in the stenotic bypass at the 3-month follow-up.

The patency of all bypasses was confirmed by ultrasonographic blood flow measurements in the 6-month observation period. The results of the ultrasonographic blood flow measurements during the observation period are shown in Figure 3. The mean ± SD systolic velocities at 1 month, 3 months, and on the day of tissue harvest were 35.37 ± 10.14, 30.56 ± 11.08, and 33.00 ± 17.31 cm/s, respectively. One bypass showed reduced blood flow velocity at 10 weeks, and a partial stenosis was observed 15 mm from the distal anastomosis. This stenotic bypass was 35.44 cm/s at 2 months but subsequently decreased to 11.73 and 9.84 cm/s at 3 months and on the day of tissue harvest, respectively. The other bypasses showed no significant reduction in blood flow or stenosis on vascular ultrasound.

**Figure 4:**
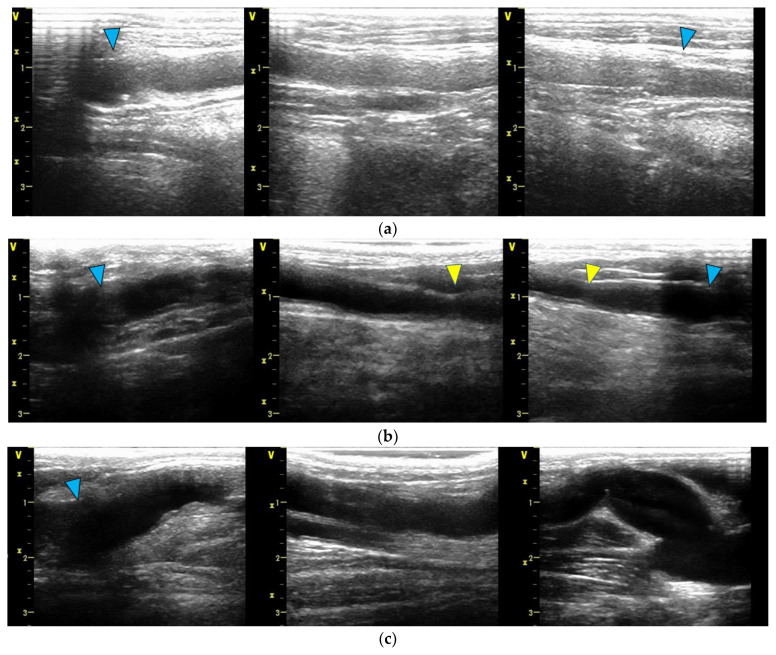
Ultrasound findings at the 6-month mark. In all diagrams, the proximal portion is depicted on the left side, and the peripheral portion is depicted on the right side. The blue arrow indicates the anastomosis site: (**a**) Four bypasses exhibited smooth luminal surfaces with no evidence of stenosis or aneurysm formation. (**b**) One bypass developed focal stenosis, as highlighted by the yellow arrow. (**c**) One bypass had a dilatation of the Biotube around the peripheral anastomosis, with a maximum diameter of 11.2 mm.

Figure 4 presents the ultrasound examination findings obtained at 6 months after the surgical procedure. In the bypass contralateral to the stenotic bypass, dilatation of the Biotube was observed near the distal anastomosis. The graft underwent gradual dilatation from week 4, reaching a maximum diameter of 11.2 mm at 13 weeks. However, no additional dilatations were observed thereafter. No other areas showed dilatation, and no ruptures or pseudoaneurysms were observed throughout the observation period.

**Figure 5:**
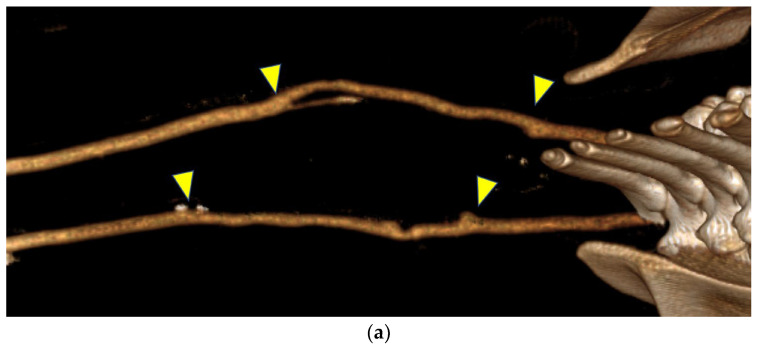

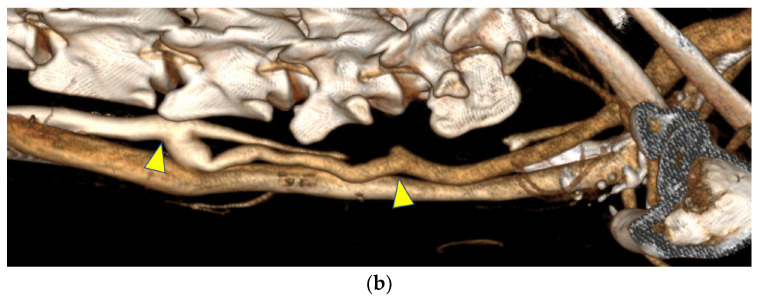
(**a**) In contrast-enhanced computed tomography, the bypasses appeared similar to the native arteries. (**b**) A bypass in one goat developed a dilatation at the peripheral anastomosis, whereas the contralateral bypass exhibited poor visualization, which was likely due to decreased blood flow. Both figures are oriented with the head on the left. The anastomosis site is indicated by the arrows.

Contrast-enhanced CT images before the tissue harvest are shown in Figure 5. The Biotube that developed stenosis, as observed on ultrasonography, was poorly visualized. Dilatation near the distal anastomosis was observed in one Biotube. No dilatations, stenoses, or pseudoaneurysms were observed in the other bypasses.

**Figure 6:**
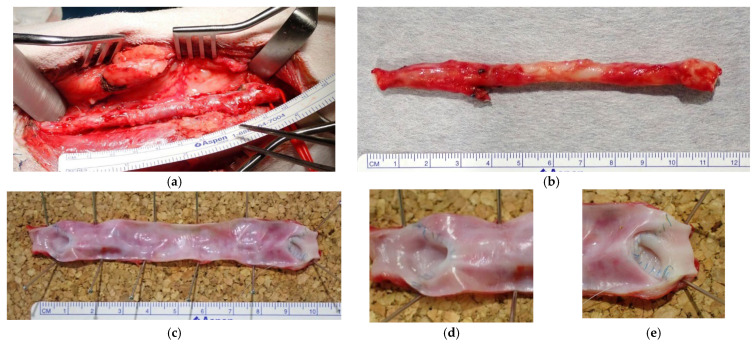

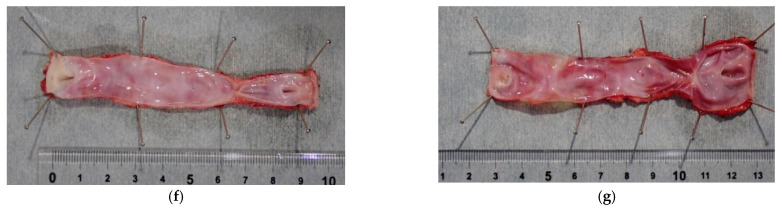
(**a**) The Biotube was loosely adherent to surrounding tissues, facilitating its dissection in a manner similar to that of native vessels. (**b**) Macroscopically, the Biotube exhibited neovascularization, closely mimicking the appearance of a native vessel. The anastomosis was visually indistinct. (**c**–**e**) The luminal surface was smooth, with no evidence of thrombosis. Both the proximal (**d**) and distal (**e**) anastomoses exhibited robust healing and a seamless transition between the Biotube and host vessel. (**f**) One bypass exhibited stenosis at 15 mm from the peripheral anastomosis. (**g**) The contralateral bypass exhibited dilatation of the Biotube in the vicinity of the peripheral anastomosis. Despite the dilatation, a continuous transition was observed at the anastomosis, and no pseudoaneurysm was identified.

After image evaluation, the Biotubes were harvested along with the carotid artery around the anastomosis. The Biotube and subcutaneous tissue were clearly demarcated, and the adhesion was loose, making the Biotubes easy to expose (Figure 6a). No inflammation was observed around the Biotube. The Biotube and carotid artery were very similar in appearance, and the anastomosis between them was unclear (Figure 6b). The mean ± SD bypass blood flow just before tissue harvest was 428.2 ± 261.5 mL/min. A longitudinal incision was made in the Biotube to observe the luminal surface. It was smooth and white like vascular endothelium throughout the Biotubes, with no evidence of thrombus or calcification (Figure 6c). At the anastomosis, the native arteries and Biotubes were adherent, and the intima was smooth and continuous (Figure 6d,e). The two Biotubes that had stenosed or dilatated were also smooth on the surface, with no differences from the other Biotubes. Stenosis occurred 15 mm from the anastomosis in the stenotic bypass (Figure 6f). The stenosis was severe but without thrombosis, and the lumen was patent. Macroscopic findings confirmed the dilatation of one Biotube (Figure 6g). Relative stenosis of the vessel proximal to the dilatated portion was observed at approximately 13 mm from the distal anastomosis. However, no wall fragility was noted in the dilatated section.

### 3.3. Histological Evaluation

**Figure 7:**
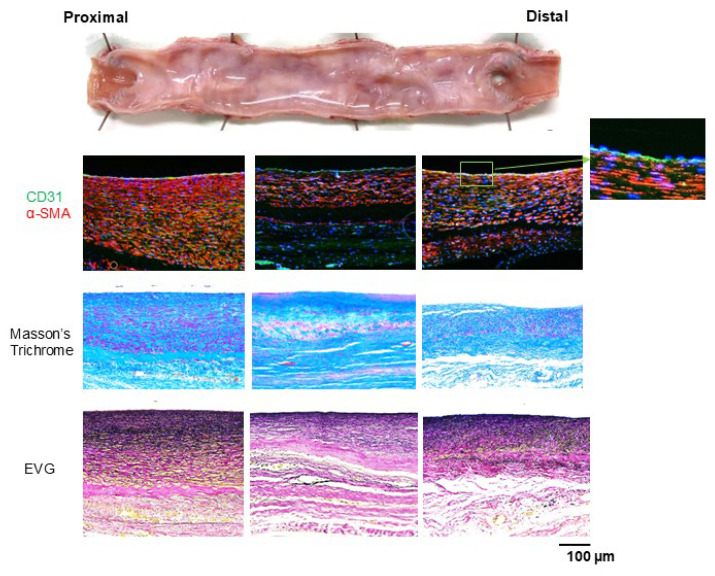
A neointima of α-SMA-positive cells (stained red) formed on the Biotube surface, with increased thickness near the anastomosis. The luminal surface was fully endothelialized, as demonstrated by CD31 positive staining (stained green). Masson’s trichrome staining revealed a collagenous layer in the Biotube that was thinner after than before implantation. Elastica van Gieson staining revealed the formation of elastic fibers within the neointima, with a higher density of elastic fibers observed closer to the lumen.

Figure 7 shows the histological findings of the Biotubes 6 months postoperatively. No infiltration of inflammatory cells or calcification was observed in any of the Biotubes. Layers of neoplastic cells were observed along both the internal and external surfaces of the Biotubes. Immunostaining revealed that α-SMA-positive cells developed in the neointima. Greater development of the neointima was observed in the vicinity of the anastomosis. Furthermore, the presence of CD31-positive cells on the intraluminal surface of the graft was identified, indicating the presence of endothelial cells. Masson’s trichrome staining confirmed the integrity of the collagen layer of the Biotube throughout the bypass. However, it was thinner than before transplantation. Elastica van Gieson staining showed the development of elastic fibers in the neointima. An elastic fiber layer was also observed on the external surface of the Biotube, but the density increased with proximity to the lumen.

**Figure 8:**
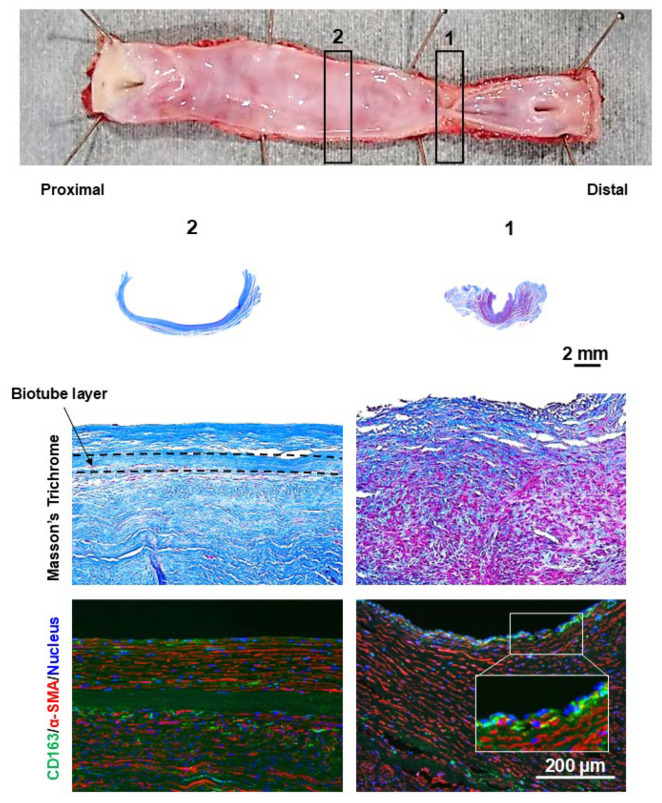
The stenotic region of the bypass graft exhibited granulation tissue formation on the luminal side. The Biotube layer (within dashed lines) was not clearly visible at the stenotic region. The granulation tissue was characterized by an overabundance of smooth muscle cells, as confirmed by α-SMA staining (stained red). CD163 staining revealed localized clusters of macrophages (stained green) on the intimal surface. The numbers of gross findings match the numbers of pathological findings.

Granular tissue formation was observed at the stenotic site of the stenotic bypass (Figure 8). At the luminal surface of the granulation tissue, focal accumulations of CD163-positive cells, consistent with macrophages, were observed. Excessive proliferation of α-SMA-positive cells was observed in the underlying layer where the macrophages were aggregated. In nonstenotic areas, CD163-positive cells were predominantly localized to the outer surface of the Biotube. Furthermore, CD163-positive cells exhibited an elongated morphology and were intercalated between α-SMA-positive cells.

**Figure 9:**
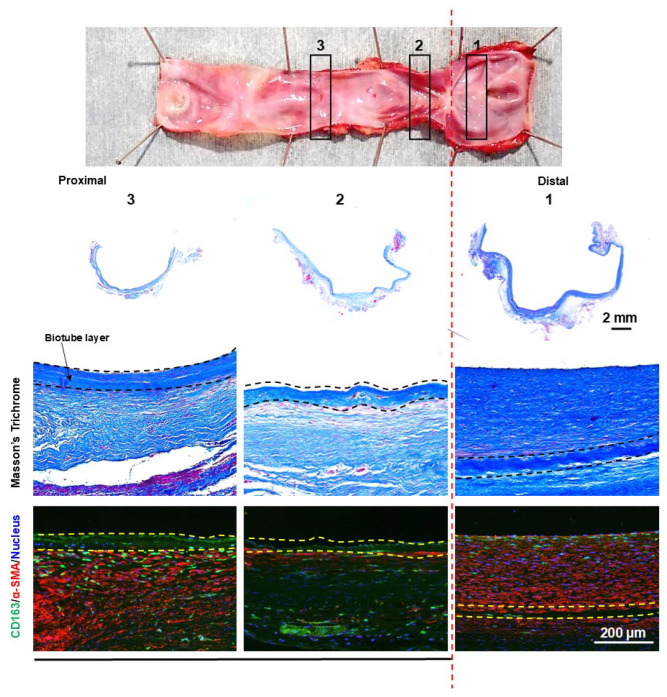
The dilatated segment of the bypass graft showed no evidence of localized thinning, with the thickness of the dilatated portion consistently being greater than 1 mm. α-SMA staining of the dilatated region revealed the presence of a neointimal layer of smooth muscle cells (stained red) on the luminal side of the Biotube (within the dashed lines). However, this neointimal layer was absent in the central, nondilatated portion of the graft. CD163 staining revealed a diffuse infiltration of macrophages (stained green) surrounding the Biotube. The numbers of gross findings match the numbers of pathological findings.

The dilatated portion exhibited no focal areas of wall thinning. Instead, it had a uniform wall thickness of over 1 mm in the dilatated bypass (Figure 9). In the dilatated segment, cellular proliferation was observed on the luminal surface of the Biotube but was absent in the central portion beyond the relatively stenotic area. Despite the absence of cellular proliferation on the luminal surface, cellular proliferation was evident on the external surface of the Biotube.

## 4. Discussion

The development of a novel, small-diameter, long graft could overcome the limitations of the saphenous vein and improve outcomes for patients with CLI requiring bypass surgery. An ideal vascular prosthesis should be biocompatible, durable, and resistant to infection. Furthermore, it should have low immunogenicity, inhibit thrombosis, and possess sufficient mechanical strength. The ideal prosthetic material would demonstrate handling qualities comparable to those of vein grafts. Furthermore, surface modifications that promote endothelial growth and long-term hemodynamic stability are essential. Although TEVGs are being explored extensively as a potential solution to these challenges, none of those developed have yet fully met all of these criteria [12,13,14,15,16].

Our institution has been at the forefront of developing cardiovascular surgical materials using iBTA technology [19,21,22]. iBTA technology is a low-complexity technique that uses the patient’s intrinsic biological response to facilitate graft production. This approach eliminates the need for specialized scaffolds, environments, or techniques, thereby streamlining the process and reducing production costs [17]. The Biotube is composed entirely of type I collagen, a natural protein known for its low immunogenicity and excellent biocompatibility [23,24,25]. Collagen can be used as a scaffold for the induction of growth factors and cells, as well as for the delivery of bioactive substances. Collagen, which promotes neointima formation and endothelial cell growth, coats the luminal surface of vascular prostheses [25,26], making them highly suitable biomaterials for TEVGs that significantly reduce the risk of immune rejection and improve long-term graft patency. Our previous studies have demonstrated the efficacy of iBTA technology in aortic valve and aortic tissue engineering, prompting us to initiate a preliminary study on developing a TEVG for peripheral arterial disease [18,19,21]. The preliminary study indicated that the Biotubes could achieve sufficient mechanical strength through 2–3 months of mold implantation while exhibiting favorable biocompatibility and handling characteristics. Furthermore, the material’s elastic properties, which were previously demonstrated to closely resemble those of native arteries, offer a significant advantage for its potential application as a vascular bypass graft [17]. In this study, we successfully maintained the patency of a 4 mm diameter Biotube for 6 months in a goat carotid bypass model, employing the same protocol as that in the preliminary study.

The regenerative process following Biotube implantation is a critical aspect of this study, providing valuable insights into the long-term efficacy and safety of this novel biomaterial. As shown in implantation studies using iBTA materials, regenerative processes ensue along the material from the aortic wall at the anastomosis site [19,21]. After implantation, the ingrowth of the host tissue from the anastomosis site along the Biotube was observed within 2–3 months. This process resulted in the formation of a neointima composed of α-SMA-positive cells. Furthermore, the collagen component of the Biotube gradually degrades, leading to its eventual replacement by the newly formed vascular tissue [18]. These findings demonstrate the favorable properties of the Biotube as a scaffold for tissue regeneration. Our findings from this 6-month implantation experiment successfully replicated the histological findings observed in the preliminary study. These results provide compelling evidence of Biotubes as promising candidates for TEVGs for the treatment of peripheral vascular disease.

Although the Biotube bypass is very promising, several technical limitations must be addressed to ensure successful outcomes. Because the structure is composed entirely of perfect collagen fibers, endothelial cells are absent. The absence of endothelial cell formation in the immediate posttransplant period prevents the inhibition of thrombosis. In our preliminary study, we found that acute thrombotic occlusion was a prevalent complication [18], which indicates the critical role of thrombosis in the development of efficacious vascular grafts [27,28]. Furthermore, goats and sheep exhibit hypercoagulability when used as animal models [13]. We had previously implemented several strategies to prevent acute graft thrombosis in the perioperative period [18]. To inhibit intraoperative graft thrombosis, we administered heparin systemically to prolong the ACT to over 300 s and avoided clamping the Biotube to prevent blood stasis. Postoperatively, the goats received an intensified anticoagulation regimen consisting of dalteparin, aspirin, and clopidogrel for 3 months. Although this regimen is a more aggressive anticoagulation strategy than that employed in standard bypass procedures, it significantly reduced the incidence of acute thrombosis. Preventing early thrombosis is a prerequisite for subsequent endothelialization.

Successfully preventing acute occlusion will facilitate the endothelialization of the luminal surface [27]. Although the regeneration of the α-SMA-positive cell layer varied between the anastomotic site and the central region, the preliminary 3-month model exhibited evident endothelialization throughout the graft [18]. Endothelialization is achieved through two primary mechanisms: the continuous migration of endothelial cells from the native arterial wall and the recruitment of endothelial progenitor cells derived from the bone marrow [29,30]. Blood-derived endothelial progenitor cells may contribute to endothelialization, thereby facilitating rapid endothelialization even in long-distance bypass grafts [27,31]. This finding was replicated in the present study, where complete endothelialization was observed at 6 months. Antiplatelet therapy was discontinued after 3 months, and the patency of the bypass graft was maintained. Therefore, long-term patency may be achieved once complete endothelialization is established.

In this study, one side of the bypass graft in one goat exhibited stenosis, while the other side demonstrated dilatation. All observed morphological changes occurred 2 cm proximal to the peripheral anastomosis. A comparable pattern was also observed in the preliminary data [18]. Excessive proliferation of smooth muscle cells and deposition of the extracellular matrix, which cause intimal hyperplasia, may be the primary cause of chronic graft stenosis, which significantly affects the long-term patency of bypass grafts [30,32]. Histological evaluation demonstrated significant accumulation of macrophages within the intimal layer of the stenotic region. Previously, we demonstrated that fibrin adhesion to the luminal surface of the Biotube occurs, followed by the accumulation of CD163-positive macrophages on this fibrin-coated surface [18]. CD163-positive macrophages contribute to both inflammatory regulation and tissue regeneration [33]. CD163-positive cells play a beneficial role in the self-organization of TEVGs through their tissue repair functions, including the secretion of vascular endothelial growth factor. CD163-positive cells on the outer surface of the Biotube may play a role in tissue regeneration. However, within the arterial environment, these macrophages exacerbate atherosclerosis in response to plaque development [34,35,36]. Despite the absence of fibrin deposition in this study, the curved regions of the bypass may be predisposed to thrombus formation or plasma protein adhesion. The subsequent recruitment of macrophages to the thrombus may stimulate the proliferation of smooth muscle cells, potentially resulting in stenosis. Inflammatory responses stimulate smooth muscle cells to produce matrix metalloproteinases, thereby promoting smooth muscle cell migration and proliferation [32]. Our previous investigation consistently demonstrated that stenosis is initiated approximately 2–3 months after implantation [18]. This temporal correlation with the completion of endothelialization within the graft lumen implies a mechanistic link between these two events. Based on these findings, acute thrombosis within the Biotube must be prevented, especially during the perioperative period. Therefore, perioperative anticoagulation and antiplatelet therapy should be administered until endothelialization is completed.

Progressive dilatation of one Biotube was observed during the first two postoperative months. No further increase in the luminal diameter was observed at subsequent time points. The observed dilatation during the acute phase may have been influenced by the reduction in initial graft strength. Although the Biotube may have adequate mechanical properties, it causes difficulty in evaluating the collagen density and strength of the graft at the implantation segment during surgery. Furthermore, a relative stenosis in the upstream segment of the dilatated area was observed in the harvested tissue. This finding indicates the potential involvement of fluid dynamics factors, such as turbulent flow, during post-stenotic dilatation [30,37]. The progression of regeneration along with increased smooth muscle cell proliferation and further reinforcement by the distribution of elastic fibers all helped inhibit further dilatation. Achieving adequate strength to resist blood pressure soon after transplantation is a critical consideration. Turbulence and shear stress may induce intimal hyperplasia, thereby requiring the careful consideration of hemodynamically stable bypass designs [30,32].

Infection during mold implantation can potentially negatively impact Biotube formation. The presence of inflammation, regardless of infection, significantly impacts the long-term patency of the bypass graft. Mold-to-mold contact can induce inflammation and infection [18]. To minimize these risks, we implanted two molds at a sufficient distance on one side of the thoracoabdominal region in this study. Although clinical settings generally emphasize wound care, thereby lowering the risk of infection compared with animal models, the implantation site requires careful consideration and strict infection control measures.

In this preclinical study, we investigated the regeneration process and long-term patency of Biotubes. However, this study has a relatively small sample size and a short observation period, which may limit the generalizability of the results. A longer observation period is essential to demonstrate the long-term patency of bypass grafts required for clinical application. Although this study demonstrated promising results in a healthy animal model, clinical patients often present with comorbidities, such as diabetes and renal failure, which may influence the biocompatibility and durability of the Biotube. The relatively short length (12 cm) and absence of joint crossing in the carotid bypass also limit the generalizability of the current findings to longer and more complex lower limb bypass procedures, such as those involving the knee joint. However, notably, a 12 cm bypass is considered relatively long in TEVG models [16]. The 4 mm graft diameter is considered to be appropriate for use in peripheral vascular bypass procedures in humans. The promising results of this study are the bases of clinical trials in human subjects currently underway (jRCT2072220062).

## 5. Conclusions

This study successfully established a 6-month goat model of a long-distance carotid bypass utilizing a 4 mm diameter Biotube. While the need for further research to explore the underlying mechanisms of bypass stenosis and dilatation was highlighted, the results indicate that Biotube demonstrates potential as an effective alternative graft for a peripheral vascular bypass.

## Data Availability

Due to the nature of this research, participants in this study did not agree to their data being shared publicly, so supporting data are not available.

## Abbreviations

PAD	Peripheral artery disease
CLI	Critical limb ischemia
TEVG	Tissue-engineered vascular graft
iBTA	In-body tissue architecture
ACT	Activated clotting time
CT	Computed tomography
α-SMA	α-smooth muscle actin
